# Adherence to hormone therapy among women with breast cancer

**DOI:** 10.1186/1471-2407-14-397

**Published:** 2014-06-03

**Authors:** Claudia Brito, Margareth Crisóstomo Portela, Mauricio Teixeira Leite de Vasconcellos

**Affiliations:** 1 Department of Health Administration and Planning of National School of Public Health Sergio Arouca, Oswaldo Cruz Foundation, Av. Leopoldo Bulhões, 1480/ Sala 717 –Manguinhos, 21041-210 Rio de Janeiro, RJ, Brazil; 2 National School of Statistical Sciences, Brazilian Institute of Geography and Statistics, Rio de Janeiro, RJ, Brazil

**Keywords:** Breast cancer, Adherence, Hormone therapy, Tamoxifen, Aromatase inhibitors, Organization of care, Risk factors, Health care organization, Quality of health care

## Abstract

**Background:**

Despite the excellent results obtained with hormone therapy, the long treatment period and the side effects associated with its use make patient adherence difficult. Moreover, certain aspects of health care can mitigate or exacerbate non-adherence. This study aimed to identify the factors associated with adherence to hormone therapy for breast cancer, with the goal of contributing to the reformulation of the care process and to improvements in outcomes.

**Method:**

This was a retrospective longitudinal study based on secondary data. The study integrated and analyzed data from a cohort of 5,861 women with breast cancer who were identified in the databases of the Brazilian National Cancer Institute [Instituto Nacional de Câncer - INCA] and the Unified Health System [Sistema Único de Saúde - SUS]. All of the patients were treated at INCA, which dispenses free medication, and the follow-up period lasted from 01/01/2004 to 10/29/2010. The outcome of interest was hormone treatment adherence, which was defined as the possession of medication, and a logistic regression model was employed to identify the socio-demographic, behavioral, clinical, and health care variables that were independently associated with the variations in this outcome.

**Results:**

The proportion of women who adhered to hormone therapy was 76.3%. The likelihood of adherence to hormone therapy increased with each additional year of age, as well as among women with a secondary or higher level education, those with a partner, those who underwent surgery, those who had more consultations with a breast specialist and clinical oncologist, and those who underwent psychotherapy; the effect for the latter increased with each additional consultation. Conversely, the likelihood of adherence was lower among patients at a non-curable stage, those who were alcohol drinkers, those who received chemotherapy, those who had undergone more tests and had more hospitalizations, and those who used tamoxifen and combined aromatase inhibitors.

**Conclusion:**

This study shows that approximately a quarter of the women with breast cancer did not adhere to hormone treatment, thus risking clinical responses below the expected standards. It also identifies the most vulnerable subgroups in the treatment process and the aspects of care that provide better results.

## Background

Studies of endocrine therapy for breast cancer treatment have been increasingly performed throughout the world [1] due to the large patient volume [2], the long treatment duration [3], the optimal obtained results [4,5], and the adverse drug effects [6,7].

Adjuvant endocrine therapy or hormone therapy for breast cancer involves the use of hormone suppressants or similar substances to inhibit tumor growth [7]. This type of therapy is associated with lower rates of disease recurrence and metastasis and, thus, improves mortality rates and disease-free survival [4,8]. Consequently, its use and related expenses have increased significantly in recent decades [2].

The duration of hormone treatment is relatively long, and patients are required to use the medication daily for 5 years [3] to obtain the maximum benefits [9]. The increase in oral medication use has highlighted a potential problem in the adherence to cancer treatment in both health and economic terms. If patients do not take their medications, they will not benefit from them, thus resulting in increased mortality and morbidity as well as increased costs and consumption of care resources. Moreover, if the doctor is unaware of the patient’s non-adherence to oral therapy, he/she might attribute the disease progression to the ineffectiveness of the drug and change the regimen unnecessarily [10] or prescribe a second- or third-line medicine with greater side effects or costs. In Brazil, breast cancer is the leading cause of cancer deaths among women (12,852 deaths in 2010), and it is estimated that in 2013, 52,680 women will be diagnosed with this type of tumor [11].

Despite the excellent results obtained with hormone therapy, the long treatment period and the side effects associated with its use make patient adherence difficult, which can lead to adverse clinical outcomes [12,13]. Moreover, certain aspects of health care (e.g., the doctor-patient relationship and the management of side effects) can mitigate or exacerbate non-adherence [1,8], even for patients with a good prognosis [14,15].

This study aimed to identify the factors associated with adherence to hormone therapy for breast cancer, with the goal of contributing to the reformulation of the care process and to improved outcomes.

## Methods

A hospital-based population retrospective longitudinal study was conducted with secondary data from women with breast cancer who received hormone therapy prescriptions from the National Cancer Institute (Instituto Nacional de Câncer; INCA) of Brazil.

INCA is the Ministry of Health reference center for cancer policies and care [16] and as such, INCA integrates the public health system and provides all inpatient and outpatient oncological treatment modalities free of charge. It is also the largest breast cancer care provider in the state of Rio de Janeiro, which has the highest incidence of this disease in Brazil [11].

The study included all women with breast cancer who appeared in the hospital-based cancer registry (hospital-based cancer registry; RHC) between 2002 and 2008 who started hormone treatment with tamoxifen (TMX) and/or the aromatase inhibitors (AIS) letrozole or anastrozole on or after 01/01/2004, and who received dispensed medication more than once before 10/29/2010, according to the INCA pharmacy (the only medicine dispensary for those women).

An integration and analysis of the information found in the following databases was performed as follows:

a. The dispensing database from the INCA pharmacy aggregated data about medicine dispensation, including the date, type (TMX, letrozole and anastrozole) and quantity. It permitted the acquisition and delivery control of medicine with satisfactory accuracy. Only patients who started hormone therapy after 01/01/2004 were considered because this database was established in October 2003 and thus included patients who required continuous treatment. The last included dispensing date was 10/29/2010.

b. The RHC database was implemented at INCA according to the recommendations of the International Agency for Research on Cancer (IARC) and was employed in the study to obtain sociodemographic and clinical variables.

The study inclusion criteria for women with breast cancer tumors who were enrolled between 2002 and 2008 relied on data availability at the time of the study. The RHC was organized by tumor, which meant that a single patient with more than 1 primary malignant tumor (excluding recurrence or metastasis) could be registered more than once. For patients with multiple recorded tumors, we used the more complete observation, the observation with the highest stage if the diagnosis dates were the same or the earliest observation if the diagnosis dates were different.

c. The Integrated Hospital System (Sistema Hospitalar Integrado; SHI) and the INCA Absolute System databases were used to record the procedures provided to women with breast cancer. The SHI was established in 1998 and was used at INCA until 2004, at which time it was replaced by the Absolute system. We evaluated data from 01/01/2002 to 10/29/2010. The Absolute system had more data categories than the SHI and thus required data cleaning to make the records compatible. The option for the use of both SHI and Absolute as sources of health care variables relied on their comprehensiveness; this was in contrast to RHC, which only included data concerning chemotherapy, radiotherapy, and oncological surgery.

After combining the databases, the differences in dates between the beginning of hormone treatment and the diagnosis of breast cancer were calculated. There were 198 cases with negative values; these were most likely due to typographical errors, and the data were subsequently corrected according to the following procedures. (1) If the initial hormone treatment date was ≤ 3 months earlier than the date of diagnosis, the diagnosis and the initiation of hormone treatment were assumed to coincide (i.e., the difference was equal to 0). (2) If the negative difference between the start of hormone therapy and the diagnosis was > 3 months, and if the second medication dispensing date was consistent with the diagnosis date, the first dispensing date was ignored and replaced with the second date, and the amount dispensed on the discarded date was deducted from the total. With these procedures, it was possible to retain 185 cases in the analysis; however, 13 women were eliminated due to a complete lack of consistency in the data.

Thus, data for 5,861 women were retained for analysis in the integrated database. There was no systematic association between the excluded cases and the variables of interest; thus, there is a low probability that data exclusion due to operational issues might have introduced bias into the study.

Adherence is defined as the extent to which a patient acts in accordance with the prescribed interval and dose of a dosing regimen, and is operationalized, in a retrospective assessment, as the number of doses dispensed in relation to the dispensing period, often called the “medication possession ratio (MPR)” [12,17-19].

It was calculated, for each woman, by summing up all quantities dispensed, and dividing the sum by her time in the cohort, that, in turn, was given by the difference between the last and the first dispensing date summed to the last dispensed quantity, for those that did not die, and between the death and the first dispensing date, for those that died [12,17-19]. We considered the recommendation of a daily hormone therapy (HT) pill for five years. The drugs that INCA dispensed free of charge were TMX and AIS.

Patients with an MPR ≥ 80% were considered adherent, which is a criterion widely applied [20-22].

The independent variables of interest included socio-demographic variables (RHC) such as the age at diagnosis, education, and marital status; clinical variables (RHC) such as the histological tumor type, stage, laterality, family history of cancer, smoking, and alcohol consumption; and treatment and health care variables (SHI/Absolute). The latter included the type of hormone therapy (e.g., TMX only, AIS only, letrozole or anastrozole, or both TMX and AIS, which indicated a switch from one form of therapy to the other), surgery, chemotherapy (CT), radiation therapy (RT), hospitalizations, clinical consultations with mastologists, clinical oncologists and other doctors, psychotherapy, multi-professional therapeutic support (MTS) (including nursing ambulatory care, nutrition services, physical therapy, speech therapy and psychology), services from a social worker, dentist or pharmacist, diagnostic and therapeutic services (DTS), and the time between the diagnosis and the initiation of hormone treatment.

Bivariate analyses of adherence were conducted with the chi-squared (χ^2^) test for categorical independent variables and Student’s t-test for continuous variables. Multivariate logistic regression was also used, while excluding variables without independent effects (p ≥ 0.15) for parsimoniousness. The analyses were performed with the SAS® statistical software package, version 9.1 (SAS Institute Inc., 2003; Cary, NC, USA).

The study was approved by the Research Ethics Committee of INCA under number 84/2010.

## Results

The patient age at diagnosis ranged from 21 to 103 years, with a mean of 57.5 (SD = 13.6) years and a median of 56.6 years. Approximately 50% of the women were between 40 and 59 years old, and only a minority was younger than 40 years. Half of the women in the cohort had a low level of education (e.g., they were illiterate or had an incomplete elementary education), and 10% had completed a higher education level.

The adherence rate was compiled from the information for each time frame as follows:

The mean and median follow-up periods were 1225 (SD = 631) and 1209 days, respectively. The cohort showed a mean and median adherence of 86.3% and 94.3%, respectively. If the patients with at least 80% adherence to treatment were assumed to be adherent, the proportion of adherent patients was 76.3%.

The group of adherent women was slightly older than the group of non-adherent women, with mean ages of 58.0 and 56.0 years, respectively (student’s t-test, p < 0.0001). Based on the chi-square test, we found associations of adherence with all categorical variables except the *histological primary tumor type* (p = 0.6049) and *laterality* (p = 0.2690; Tables 1, 2 and 3).

**Table 1:** Distribution of socio-demographic characteristics according to adherence to hormone treatment (N = 5,861)

**Variable**		**Adherent**		**Non-adherent**	**χ**^**2 **^**(p)**
	**N**	**%**	**N**	**%**	
**Age (years)**					<0.0001
< 40	342	65.9	177	34.1	
40 - 49	1,052	74.4	362	25.6	
50 - 59	1,165	77.7	334	22.3	
60 - 69	950	79.0	253	21.0	
> 70	961	78.4	265	21.6	
**Education**					0.0530
Illiterate + incomplete 1st grade	2,219	75.4	724	24.6	
Complete 1st grade	772	74.8	260	25.2	
2nd grade + higher education	1,444	78.5	395	21.5	
No information	35	74.5	12	25.5	
**Relationship status at start of treatment**					0.0022
With a partner	2,135	78.3	590	21.6	
Without a partner	2,307	74.5	791	25.5	
No information	28	73.7	10	26.3	

**Table 2:** Distribution of clinical characteristics according to adherence to hormone treatment (N = 5,861)

**Variable**		**Adherent**	**Non-adherent**					**χ**^**2 **^**(p)**
	**N**	**%**	**N**	**%**				
**Family history of cancer**					0.0454
Yes	2,519	77.5	732	22.5	
No	1,816	74.9	610	25.1	
No information	135	73.4	49	26.6	
**Alcohol consumption**					0.0082
Yes	1,181	73.6	424	26.4	
No	3,164	77.4	924	22.6	
No information	125	74.4	43	25.4	
**Smoker**					0.0014
Yes	1,503	73.9	530	26.1	
No	2,902	77.7	832	22.3	
No information	65	69.1	29	30.8	
**Histological type of primary tumor**					0.6049
Invasive ductal carcinoma (IDC)	3,561	76.1	1117	23.9	
Other tumors	909	76.8	274	23.2	
**Laterality**					0.2690
Unilateral	4,293	76.4	1325	23.6	
Bilateral	173	73.3	63	26.7	
No information	4	57.1	3	42.8	
**Stage**					< 0.0001
Curable	2,742	83.4	544	16.6	
Non-curable	1,549	65.3	822	34.7	
No information	179	87.7	25	12.2	

**Table 3:** Distribution of breast cancer treatment procedures according to adherence to hormone treatment (N = 5,861)

**Variable**		**Adherent**		**Non-adherent**	**χ**^**2 **^**(p)**
	**N**	**%**	**N**	**%**	
**Type of hormone therapy**					<0.0001
TMX only	3,014	79.8	762	20.2	
AIS only	256	75.5	83	24.5	
Both (TMX + AIS)	1,200	68.7	546	31.3	
**Surgery**					0.0007
Yes	2,720	77.8	775	22.2	
No	1,750	74.0	616	26.0	
**Chemotherapy**					< 0.0001
Yes	2,553	72.3	978	27.7	
No	1,917	82.3	413	17.7	
**Radiotherapy**					< 0.0001
Yes	2,036	73.4	736	26.5	
No	2,434	78.8	655	21.2	
**Therapeutic combination**					< 0.0001
HT only	447	80.9	105	19.0	
HT and surgery	762	84.8	137	15.2	
HT and CT	395	68.2	184	31.8	
HT and RT	405	80.5	98	19.5	
HT, CT and surgery	830	78.4	229	21.6	
HT, RT and surgery	303	80.6	73	19.4	
HT, CT and RT	503	68.7	229	31.3	
HT, CT, RT and surgery	825	71.1	336	28.9	
**CT frequency**					< 0.0001
No procedure	1,917	82.3	413	17.7	
1 to 3 procedures	560	81.2	130	18.8	
4 to 6 procedures	1,463	82.3	314	17.7	
≥ 7procedures	530	49.8	534	50.2	
**Frequency of hospitalization**					< 0.0001
None	752	78.2	209	21.7	
One	2,250	80.5	544	19.5	
Two	909	76.9	273	23.1	
≥ 3	559	60.5	365	39.5	
**Mastologist consultations**					< 0.0001
None	343	55.6	274	44.4	
1 to 4 consultations	693	69.2	308	30.8	
5 to 13 consultations	2,473	83.2	498	16.8	
≥14 consultations	961	75.5	311	24.5	
**Clinical oncologist consultations**					< 0.0001
None	1,006	80.3	251	20.0	
1 to 4 consultations	1,325	79.1	349	20.8	
5 to 12 consultations	1,126	74.6	384	25.4	
≥13 consultations	1,013	71.3	407	28.7	
**Other physician consultations**					< 0.0001
≤ 9 consultations	410	67.9	194	32.1	
10 to 22 consultations	1,683	78.0	475	22.0	
23 to 34 consultations	1,316	80.5	319	19.5	
≥35 consultations	1,061	72.5	403	27.5	
**Psychotherapy consultations**					< 0.0001
None	2,422	72.7	909	27.3	
1 to 3 consultations	1,577	80.0	394	20.0	
≥4 consultations	471	84.3	88	15.7	
**Therapeutic support consultations**					< 0.0001
None	994	70.8	410	29.2	
1 to 3 consultations	1,553	77.3	457	22.7	
4 to 7 consultations	872	77.2	258	22.8	
≥8 consultations	1,051	79.8	266	20.2	
**DATS (tests)**					< 0.0001
None	870	78.2	242	21.8	
1 test	1,587	79.1	420	20.9	
2 to 3 tests	1,377	75.8	439	24.2	
≥4 tests	636	68.7	290	31.3	

Table 1 presents the results according to sociodemographic variables and shows the lower likelihood of adherence among younger women (<40 years). Table 1 also shows the higher likelihood of adherence among women who had completed second grade or higher and among those with a partner; these factors were found to have borderline associations.

In relation to the clinical variables (Table 2), the results show a higher likelihood of adherence among women with a family history of cancer and a lower likelihood of adherence among women who were alcohol drinkers or smokers. The likelihood of adherence was especially low among those who were diagnosed with cancer at a non-curable stage (III and IV).

With regard to the variables related to patient care (Table 3), there was a higher likelihood of adherence in women who were treated with TMX alone, as well as those who underwent surgery. In contrast, women who were treated with both types of hormone therapy (switching from 1 form of therapy to the other), who underwent radiotherapy, who had more consultations with clinical oncologists, or who underwent seven or more chemotherapy procedures, hospitalizations, and tests all had a lower likelihood of adherence. The results also revealed a lower frequency of adherence among women who had not consulted with a mastologist and among those who had not received professional therapeutic support or psychotherapy.

Table 4 shows the multiple logistic regression model that identified the effects of the explanatory variables on adherence to hormone therapy.

**Table 4:** Multiple logistic regression model for adherence to hormone treatment (N = 5,861)

**Variable**	**Coefficient**	**Standard error**	**Pr > χ2**	**Odds ratio**	**95% CI**
Intercept	−0.3642	0.2591	0.1598		
Age^*^ (years)	0.0117	0.0029	<0.0001	1.012	1.006 – 1.018
Education					
High school diploma	0.2216	0.0858	0.0098	1.248	1.055 – 1.477
Bachelor’s degree	0.3087	0.1197	0.0099	1.362	1.077 - 1.722
Conjugal status - with a partner	0.2341	0.0681	0.0006	1.264	1.106 – 1.444
Disease stage - non-curable	−0.7329	0.0711	<0.0001	0.481	0.418 – 0.552
Alcohol consumption (yes)	−0.2266	0.0735	0.0020	0.797	0.690 – 0.921
Breast cancer surgery (yes)	0.2478	0.0686	0.0003	1.281	1.120 – 1.466
Chemotherapy (yes)	−0.2686	0.0816	0.0010	0.764	0.651 – 0.897
Hospitalizations (≥3)	−0.5322	0.0842	<0.0001	0.587	0.498 – 0.693
Mastologist visits					
1-4	0.4215	0.1135	0.0002	1.524	1.220 – 1.904
≥ 5	1.2805	0.1081	<0.0001	3.598	2.911 – 4.447
Oncologist visits					
1-4	0.2446	0.0989	0.0134	1.277	1.052 – 1.550
≥ 5	0.3203	0.0958	0.0008	1.378	1.142 – 1.662
Diagnostic and therapeutic services					
≤1 exam	0.2557	0.0788	0.0012	1.291	1.107 – 1.507
≥ 4 exams	−0.3284	0.0968	0.0007	0.720	0.596 – 0.871
Psychotherapy^*^ (consultations)	0.0534	0.0147	0.0003	1.055	1.025 – 1.086
Type of hormone therapy - both (TMX + AIS)	−0.3148	0.0751	<0.0001	0.730	0.630 – 0.846

CI = Confidence interval. *Continuous variables. References for categorical variables: education – incomplete high school or lower, no information; conjugal status at start of treatment – without a partner, no information; disease stage – curable, no information; alcohol consumption – no, no information; breast cancer surgery – no; chemotherapy – no; hospitalization – <3; mastologist consultations – none; clinical oncologist consultations – none; DTS – 2–3 exams; type of hormone therapy - TMX only, AIS only.

The likelihood of adherence increased with increasing age (OR = 1.012, 95% CI = 1.006-1.018) and with the number of psychotherapy consultations (OR = 1.055, 95% CI = 1.025-1.086). It was greater among women with a mid-level education (OR = 1.248, 95% CI = 1.055-1.477) or higher education (OR = 1.362, 95% CI = 1.07-1.722), as well as among those with a partner (OR = 1.264, 95% CI = 1.106-1.444), those who underwent breast cancer surgery (OR = 1.281, 95% CI = 1.120-1.466), those who consulted with a mastologist (1–4 visits, OR = 1.524, 95% CI = 1.220-1.904; ≥5 visits, OR = 3.598, 95% CI = 2.911- 4.447) or an oncologist (1–4 visits, OR = 1.277, 95% CI = 1.052-1.550; ≥ 5 visits, OR = 1.378; 95% CI = 1.142-1.662), and those who underwent one or no exams (OR = 1.291, 95% CI = 1.107-1.507). On the other hand, the likelihood of adherence was lower among patients diagnosed at a non-curable stage (OR = 0.481, 95% CI = 0.418-0.552), those who were alcohol drinkers (OR = 0.797, 95% CI = 0.690-0.921), those who received chemotherapy (OR = 0.764; 95% CI = 0.651-0.897), those who had three or more hospitalizations (OR = 0.587, 95% CI = 0.498-0.693) or at least four exams (OR = 0.720, 95% CI = 0.596-0.871), and those who were treated with both TMX and AIS (OR = 0.730, 95% CI = 0.630-0.846).

## Discussion

The observed level of adherence to hormone therapy (76.3%) in this study was consistent with those found in other published studies, that indicated, considering the same criteria to define adherence, proportions of 75% [20] and 72% [22], or a mean adherence of 93% [12]. However, we underline the difficulties of correlating some available previous studies due to differences in adherence definitions, eligibility criteria (e.g., patients with early tumors or only either young or elderly women), analysis methods, and the types of drugs used (only TMX, only IAS, or both).

The method employed in this study, as in others, accounts for medication delivery as a proxy for medication use, what may result in an overestimation of adherence rates. It is a limitation, but it is believed that the bias can be attenuated when estimations are made on the basis of secondary data from large populations [23].

Another limit of this study was the absence of individual information regarding treatment side effects, which could certainly affect hormone therapy adherence [24].

The truncation of adherence in cases where it reached levels above 100% is justified by the awareness of situations such as the occurrence of deaths, change of medication, and anticipated drug deliveries to meet patient needs.

Only women who received drugs at least twice were included in the study, once the MPR formula requires two dispensing dates. This procedure could have contributed to over or underestimations of the adherence rates, since who received the medication only one time could have been adherent or not to treatment in another place.

Moreover, we underline the difference between the concepts of treatment adherence and persistence, indicating that adherent patients may be non-persistent if they interrupt the treatment before the recommended period of five years.

This study was based on data from a single hospital, and we acknowledge that this might be a limitation. Nevertheless, it is important to emphasize that the hospital allows universal access, restricting entrance only to patients who was treated previously in other locations.

The observed association between adherence and age in this study agrees with some studies [12,19,21,25] yet contradicting others [26]. It can be speculated that the observed lower adherence rate among younger patients is related to the adverse effects of hormone therapy on the women’s sexuality, which include fertility issues and menopausal symptoms [13]. Moreover, the association of greater treatment compliance with having a partner, also found in other studies [22,26], might be linked to support reception. This result is consistent with the idea that social support is highly predictive of adherence [1].

In this cohort, there was greater adherence among women with higher education levels, contrasting to findings of Wigertz *et al.*[26], who did not identify a statistically significant association between adherence and education. Anyway, it must be noted that it is very difficult to ensure that education in the Brazilian context is comparable to that of developed countries, and there are few other national studies on the subject. It is plausible the observation of greater adherence among patients with higher education while considering, among other things, that socioeconomic status has also been identified as predictive of adherence [21].

With regard to the disease stage, the observation of lower compliance among patients at non-curable stages (III and IV) contradicts Wigertz *et al*. [26], who found greater adherence among women with larger tumors. Kimmick *et al*. [20] found no association between these variables. Importantly, however, the majority of studies on this topic were based only on cohorts of women with early stage disease, which reflects the reality in developed countries, wherein 60% of tumors are diagnosed at a localized stage and thus women who are fighting the disease are at a great advantage.

In Brazil, despite the efforts of the Ministry of Health to implement national policies for earlier breast cancer detection, the proportion of advanced-stage diagnoses is very high, compared to that in developed countries [27]. The lower adherence rates among women with a non-curable stage at diagnosis emphasize the importance of investments in early diagnosis since it has a direct effect on patient survival [4,8] and also an indirect effect by facilitating treatment compliance.

Bivariate analyses conducted in this study identified the association between both smoking and alcohol consumption and lower treatment adherence [21], while the multivariate analysis ratified only the effect of alcohol consumption on the dependent variable.

This study reinforces the importance of surgery in breast cancer treatment. Previously, surgery had been associated with longer survival [27,28], and herein, it was associated with greater patient adherence to hormone therapy. The current study also revealed that patient monitoring by mastologists and oncologists had a positive association effect on hormone therapy adherence [19]. However, a more intensive use of health care resources such as chemotherapy, exams and hospitalizations appeared to associate with lower adherence. This result was not directly observed in other studies but may be indirectly related to the association between lower adherence and comorbidities, observed in other studies [15,20]. Additionally, it should be emphasized that the independent effects of these clinical variables, even after controlling for the disease stage at diagnosis, might reflect the different levels of tumor aggressiveness, different levels of treatment responses, and the presence of comorbidities.

Another notable finding in this study was the independent positive effect of psychotherapy consultations on the likelihood of treatment adherence. This result might be related to those of studies that related psychological problems, particularly depression, to lower treatment adherence [1].

For Kim & Toge [29], a multidisciplinary approach to breast cancer treatment broke down barriers to access, facilitated cooperation between doctors to produce better treatment proposals, and improved the quality of care and patient satisfaction. Although the multidisciplinary team approach to oncology is required by the Brazilian Ministry of Health [16] and is a necessary condition to hospital certification for the provision of oncological care in the Unified Health System (Sistema Único de Saúde; SUS), the present study indicates that between 24.0% and 57.0% of women who were treated at INCA had no records of multidisciplinary therapeutic support consultations or psychotherapy, respectively. Thus, more consultations with nurses, psychologists, social workers and physiotherapists should be encouraged in the clinical monitoring phase. Steps that facilitate the patient’s understanding of the need for treatment and that promote and monitor adherence should be valued. Multidisciplinary therapeutic support consultations were associated with greater adherence to hormone therapy in the bivariate analysis, but were not significant in the multivariate model when psychotherapy consultations were considered separately. The role of such consultations should be further explored in other studies, which may provide more information about their effects and indicate potential changes in their objectives and uses.

With regard to the type of hormone therapy, the poor adherence among patients who used both TMX and AIS has been corroborated in others studies [19,26]. This finding might be due to the frequent musculoskeletal toxicity associated with AIS [30]. Other studies have found no statistically significant differences between the different types of hormone therapy and adherence [31,32], although a lower adherence rate has been noted for anastrozole than for TMX [32]. It is noteworthy that in the current study cohort, the majority (64.4%) of the women were treated with TMX alone, while 29.8% received a combination of TMX and AIS and a few women (5.8%) were treated only with AIS. The indication of each drug according to the patient’s morbidity profile was not analyzed.

## Conclusion

In this cohort, approximately 25% of the breast cancer patients were considered to be non-adherent to hormone treatment, thus risking a clinical response below the expected standards. Moreover, this study identified the patient characteristics that are associated with greater vulnerability while receiving hormone therapy for breast cancer and reveals the health care practices that facilitate adherence to treatment. Interventions based on these results may help improve patient adherence, quality of care and outcomes.

## Abbreviations

AIS: Aromatase inhibitors; CT: Chemotherapy; DTS: Diagnostic and therapeutic services; HT: Hormone therapy; INCA: National cancer institute; MPR: Medication possession ratio; MTS: Multi-professional therapeutic support; OR: Odds ratio; RHC: Hospital-based cancer registry; RT: Radiotherapy; SHI: Integrated hospital system; SUS: Unified health system; TMX: Tamoxifen.

## Competing interests

The authors declare that they have no competing interests.

## Authors’ contributions

All authors participated in study design, dataset preparation by combining the databases, statistical analysis, and drafting and/or reviewing the manuscript. All authors read and approved the final manuscript.

## Pre-publication history

The pre-publication history for this paper can be accessed here:

http://www.biomedcentral.com/1471-2407/14/397/prepub
